# Complete mitochondrial genome of *Cladosporium cladosporioides* YFCC 8621 isolated from a salt mine in Yunnan, southwestern China

**DOI:** 10.1080/23802359.2019.1710287

**Published:** 2020-01-14

**Authors:** Yanfang Liu, Guodong Zhang, Yao Wang, Kongfu Zhu, Wusong Yang, Yuanbing Wang, Hong Yu

**Affiliations:** a Yunnan Herbal Laboratory, School of Life Sciences, Yunnan University, Kunming, Yunnan, China;; b The International Joint Research Center for Sustainable Utilization of Cordyceps Bioresources in China and Southeast Asia, Yunnan University, Kunming, Yunnan, China;; c Nuodeng Ham Food Factory, Yunlong, Yunnan, China;; d The Research Center of Cordyceps Development and Utilization of Kunming, Yunnan Herbal Biotech Co. Ltd, Kunming, Yunnan, China

**Keywords:** *Cladosporium cladosporioides*, mitochondrial genome, phylogenetic analysis

## Abstract

*Cladosporium cladosporioides* is one of the most isolated species in the genus *Cladosporium* and has a wide medical and industrial usage. Here, we first report the complete mitogenome of *C. cladosporioides* based on the Illumina sequencing data. The circular mitogenome is 36,768 bp in length, containing 14 protein-coding genes (PCGs), 2 ribosomal RNA (rns and rnl) genes, 2 ORFs (ORF214 and ORF240), and a set of 28 transfer RNA (tRNA) genes. The overall base composition is 35.7% A, 34.0% T(U), 15.3% C, 15.0% G, with a GC content of 30.3%. Phylogenetic analysis shows that *C. cladosporioides* is clustered in the order Capnodiales and is closely related to the congeneric species *Cladosporium zixishanense* of Cladosporiaceae.

The genus *Cladosporium*, belonging to Cladosporiaceae (Capnodiales, Dothideomycetes), was built by Link in 1815. Species of *Cladosporium* occur on various hosts or substrates and are most frequently isolated from outdoor and indoor environments, other spoiled organic matters and are considered as food contaminants (Dixon and Polak-Wyss 1991; De Hoog et al. 2000; San-Martin et al. 2005). *Cladosporium cladosporioides* is one of the most isolated species in the genus *Cladosporium* as plant and animal pathogens that are ubiquitous in the environment. Additionally, *C. cladosporioides* has a wide medical and industrial usage owing to the existence of bioactive compounds, enzymes, and chlorpyrifos hydrolase (AlMatar and Makky 2016). The *C. cladosporioides* complex has been widely examined and phylogenetically analyzed on the basis of multiple nuclear genes (Bensch et al. 2010). However, the genomic information about this important fungus *C. cladosporioides* is lacking. This work aims to report the complete mitogenomic characterization of *C. cladosporioides* assembled from Illumina sequencing data and to validate its phylogenetic position in Capnodiales.

*Cladosporium cladosporioides* strain YFCC 8621 used in this study was isolated from a salt mine in Yunlong county of Yunnan, southwestern China (25°53′13.54′′N, 99°22′07.10′′E, alt. 1639 m). The strain YFCC 8621 was deposited at the Yunnan Fungal Culture Collection (YFCC), Yunnan University. Mycelia on PDA plates at 25 °C for 2 weeks were prepared to extract total genomic DNA using DNeasy Plant Genomic DNA Purification Mini Kit (QIAGEN). The whole-genome sequencing was carried out by Novogene Co., Ltd. (Beijing, China) on the Illumina sequencing platform (HiSeq-PE150). The software SPAdes v. 3.11.0 was used to assemble the complete mitogenome of *C. cladosporioides* (Bankevich et al. 2012). The mitogenome was annotated using MFannot tool and ARWEN web server, combined with artificial correction technology. The Organellar Genome DRAW tool was used to draw the mitogenomic circular map (Lohse et al. 2007).

The complete mitogenome sequence of *C. cladosporioides* was submitted to GenBank database under accession number no. MN 661341. The circular mitogenome is 36,768 bp in length, containing 14 protein-coding genes (PCGs), 2 ribosomal RNA (rns and rnl) genes, 2 ORFs (ORF214 and ORF240), and a set of 28 transfer RNA (tRNA) genes. The total length of the 14 PCGs (*atp6*, *8–9*, *cob*, *cox1–3*, *nad1–6*, and *nad4L*) is 12,893 bp. The lengths of 28 transfer RNA (tRNA) genes are ranging from 71 to 88 bp, and the sizes of rns and rnl are 1648 bp and 3652 bp, respectively. The sizes of ORF214 and ORF240 are 645 bp and 723 bp, respectively. The overall base composition of *C. cladosporioides* YFCC 8621 is as follows: 35.7% A, 34.0% T(U), 15.3% C, 15.0% G, with a GC content of 30.3%.

Mitogenomic sequences of 22 species in Ascomycota were downloaded from NCBI. The 14 PCGs were aligned using MUSCLE (Edgar 2004). The phylogenetic tree was constructed on the base of Bayesian inference (BI) method with the software MrBayes v.3.1.2 (Ronquist and Huelsenbeck 2003). The BI analysis was run on the MrBayes v.3.1.2 for 5 million generations using the GTR + G + I model. The phylogenetic tree showed that *C. cladosporioides* is clustered in the order Capnodiales and is closely related to the congeneric species *Cladosporium zixishanense* of Cladosporiaceae (Figure 1).

**Figure 1.**
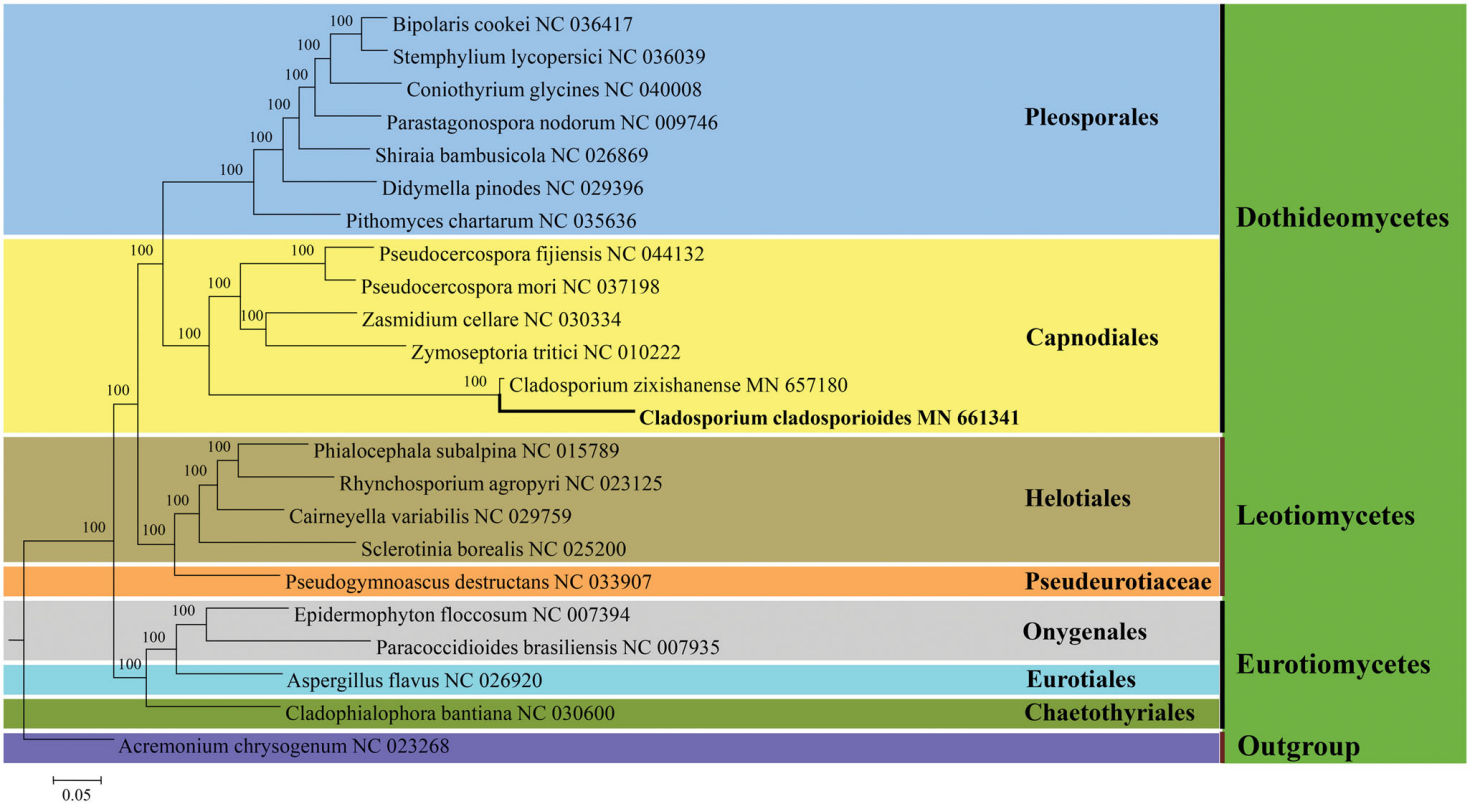
Phylogenetic relationships among 23 taxa in Ascomycota based on BI analysis from 14 concatenated mitochondrial protein-coding genes (PCGs). The BI posterior probabilities are shown above internodes.
